# Enhancing Cone-Beam CT Image Quality in TIPSS Procedures Using AI Denoising

**DOI:** 10.3390/diagnostics14171989

**Published:** 2024-09-09

**Authors:** Reza Dehdab, Andreas S. Brendlin, Gerd Grözinger, Haidara Almansour, Jan Michael Brendel, Sebastian Gassenmaier, Patrick Ghibes, Sebastian Werner, Konstantin Nikolaou, Saif Afat

**Affiliations:** Department of Diagnostic and Interventional Radiology, University Hospital Tübingen, D-72076 Tuebingen, Germany

**Keywords:** TIPSS (Transjugular Intrahepatic Portosystemic Shunt), cone-beam computed tomography, AI denoising, image quality analysis, radiation dose reduction

## Abstract

*Purpose:* This study evaluates a deep learning-based denoising algorithm to improve the trade-off between radiation dose, image noise, and motion artifacts in TIPSS procedures, aiming for shorter acquisition times and reduced radiation with maintained diagnostic quality. *Methods:* In this retrospective study, TIPSS patients were divided based on CBCT acquisition times of 6 s and 3 s. Traditional weighted filtered back projection (Original) and an AI denoising algorithm (AID) were used for image reconstructions. Objective assessments of image quality included contrast, noise levels, and contrast-to-noise ratios (CNRs) through place-consistent region-of-interest (ROI) measurements across various critical areas pertinent to the TIPSS procedure. Subjective assessments were conducted by two blinded radiologists who evaluated the overall image quality, sharpness, contrast, and motion artifacts for each dataset combination. Statistical significance was determined using a mixed-effects model (*p* ≤ 0.05). *Results:* From an initial cohort of 60 TIPSS patients, 44 were selected and paired. The mean dose-area product (DAP) for the 6 s acquisitions was 5138.50 ± 1325.57 µGy·m^2^, significantly higher than the 2514.06 ± 691.59 µGym^2^ obtained for the 3 s series. CNR was highest in the 6 s-AID series (*p* < 0.05). Both denoised and original series showed consistent contrast for 6 s and 3 s acquisitions, with no significant noise differences between the 6 s Original and 3 s AID images (*p* > 0.9). Subjective assessments indicated superior quality in 6 s-AID images, with no significant overall quality difference between the 6 s-Original and 3 s-AID series (*p* > 0.9). *Conclusions:* The AI denoising algorithm enhances CBCT image quality in TIPSS procedures, allowing for shorter scans that reduce radiation exposure and minimize motion artifacts.

## 1. Introduction

Portal hypertension (PH) is defined by an elevation in pressure within the portal venous system, mainly due to the consequences of chronic liver disease (CLD). Cirrhosis stands out as the principal cause of PH, leading to a variety of subsequent complications [1].

The implementation of the TIPSS has emerged as a fundamental approach to managing PH-related complications [2,3,4,5].

In recent years, CBCT has been identified as an innovative technique in guiding the TIPSS procedure, enabling cross-sectional imaging via a c-arm equipped with a flat-panel detector [6].

A notable limitation impeding the extensive adoption of CBCT is the significant radiation exposure it involves [7,8]. Various protocols for image acquisition exist within CBCT applications. The primary distinctions among these protocols pertain to the radiation dose and acquisition duration. However, nuances also exist regarding the positioning of the C-arm, the frame rate per rotation, and the field of view (FOV) [9]. Shortening acquisition times to reduce radiation doses can potentially result in fewer respiratory motion artifacts. While this aspect may improve certain image characteristics, the concurrent reduction in radiation dose might lower the contrast-to-noise ratio (CNR), posing a risk of generating non-diagnostic images. On the other hand, longer acquisition times may increase the likelihood of motion artifacts and are associated with a certain increase in radiation exposure. Recent advances in artificial intelligence (AI) have introduced post-processing techniques capable of augmenting image quality in radiological interventions, including scenarios affected by high body mass index (BMI) [10]. Its implementation is expected to facilitate diagnostic scans with shortened acquisition times and decreased radiation exposure, concurrently decreasing motion artifact incidence and enhancing image quality. However, the potential effects of applying deep learning to enhance cone-beam CT images during TIPSS procedures have not been thoroughly investigated. We hypothesized that the algorithm could optimize the balance between radiation dose, acquisition time, motion artifact, and image quality. This study aims to investigate the impact of a novel AI-based denoising algorithm on CBCT imaging in the context of TIPSS.

## 2. Methods

### 2.1. Study Population and Patient Characteristics

The institutional review board approved our single center’s retrospective eligibility analysis and data collection for patients undergoing interventional radiology procedures with CBCT from 1 January 2018 to 1 January 2022, including a waiver for the need for informed consent. Patients were categorized based on the image acquisition duration following a review of 8622 CBCT examinations, from which 8554 examinations unrelated to TIPSS were excluded. This resulted in 22 patients undergoing a 3 s acquisition time. Initially, 48 patients had 6 s acquisition. To ensure a matched comparison based on BMI, 24 of these with no matches were excluded, resulting in a final group of 22 patients with 6 s acquisition.

For each patient, clinical indications for TIPSS, demographics, and BMI (in kg/m^2^) were documented. Based on their BMI, patients were paired, and each pair was comparatively evaluated for both subjective and objective image qualities.

### 2.2. Image Acquisition, Reconstruction, and Postprocessing

All TIPSS procedures were executed by a senior interventional radiologist with extensive experience exceeding six years in the domain of interventional radiology. Each procedure was conducted under general anesthesia following a preliminary paracentesis. Utilizing a 10 French sheath, access to the right hepatic vein was established via the right jugular vein. Subsequent to this, the mapping of portal vein branches was facilitated through CBCT in an arms-down position. The portal vein puncture was executed under 3D guidance and successful penetration was confirmed by aspirating blood through the TIPSS needle, followed by the administration of contrast media. A standard TIPSS stent graft (Viatorr, W.L. Gore, Flagstaff, AZ, USA) was employed to secure the TIPSS tract, with the criterion for technical success being the successful delivery of contrast into the portal vein via a 4F diagnostic catheter, aiming to reduce the portosystemic gradient to 10 mmHg or below.

The images were acquired with a multiaxis robotic angiographic C-arm suite (Artis Zeego Q, VE40 A, Siemens Healthineers, Forchheim, Germany). Adherence to a standardized contrast injection protocol was maintained across all procedures, facilitated through peripheral vein access utilizing an 18G needle. The injection protocol comprised the venous administration of a diluted contrast medium (75 mL of Ultravist at 370 mg/mL; Bayer Vital GmbH, Berlin, Germany), succeeded by a saline flush delivered at a flow rate of 4.5 mL/second by an automated power injector. Initiation of the C-arm CT system was automated and triggered 60 s post-injection.

The decision between utilizing a rotation time of either three seconds or six seconds for the CBCT system was determined by the operating radiologist’s aim to precisely target the portal vein. This approach allowed for the acquisition of either 397 projection images over six seconds or 167 projection images in three seconds, adhering to a detector entrance dose of 0.36 µGy/frame and capturing images at 60 frames per second. Image acquisition was completed over a 200° circular trajectory, with the CBCT images being processed using the breath-holding technique to ensure image stability and clarity. Image data were then transmitted to an offline workstation (syngo XWP, Siemens Healthcare, Singapore). CT-like axial images with a 1 mm slice thickness and interval were reconstructed using a weighted filtered back-projection technique supplemented with a median filter. Post-processing was carried out using the AID algorithm, which is based on a modified U-net-type convolutional neural network model (CNN) (ClariCT.AI ver 1.2.1, ClariPi Inc., Seoul, Republic of Korea) [11].

### 2.3. Objective Image Quality

The images were analyzed using FIJI (ver. 1.53k, Wayne Rasband, National Institutes of Health-NIH, Bethesda, MD, USA) [12]. ROIs (Regions of interest) were drawn in key regions for the TIPSS procedure, targeting the right hepatic vein and the portal vein, as well as in intraperitoneal fat to measure the differential of CT numbers in Hounsfield Units (HU), indicative of contrast. Analysis was expanded to the AID series, with consistent placement for measuring mean CT numbers in HU and their standard deviation (SD), with SD representing image noise. Additionally, the Contrast-to-Noise Ratio for each ROI was calculated to quantitatively assess objective image quality.

### 2.4. Subjective Image Quality

For the subjective image quality evaluation, two radiologists, both with five years of experience in interventional radiology and cone-beam CT, independently reviewed all combinations of the datasets. Each pair consisted of four image reconstructions (two from 3 s acquisitions and two from 6 s acquisitions), each processed with both Original and AID methods. Utilizing ViewDex (ver. 3.0), a software engineered for blinded and randomized evaluations, the radiologists conducted their assessments across four subjective criteria: overall subjective image quality, sharpness, motion artifact burden, and contrast. Each image was rated against its pair as being of superior (+1), equivalent (0), or inferior (−1) quality. This evaluative framework, emphasizing overall subjective quality, motion artifact burden, sharpness, and contrast, was strategically chosen for its relevance in affecting the identification of the portal vein during the TIPSS procedure.

### 2.5. Statistical Analysis

Statistical evaluations and visual presentations were conducted using GraphPad Prism version 9.3.1 for the Windows platform (GraphPad Software, based in San Diego, CA, USA). The Shapiro–Wilk test was applied to assess the normality of data distribution. Means ± standard deviations represented normally distributed variables, while medians and interquartile ranges described those that were not normally distributed. The Wilcoxon signed-rank test was utilized to evaluate differences in radiation doses across different acquisition times. Additionally, Spearman’s rank correlation coefficient was calculated to assess the relationship between radiation dose and acquisition time. The significance of the interactions was determined using a one-tailed test, with *p*-values less than 0.05 considered statistically significant. Mixed-effects models, with adjustments for sphericity violations using the Greenhouse–Geisser correction, were utilized for subjective and objective image quality analysis. For objective image quality assessments, variables such as contrast, standard deviation, and CNR across different acquisition times (6 s and 3 s) and reconstruction modes (Original and AID) were analyzed as repeated measures. Subjective evaluations, including quality, motion artifact burden, sharpness, and contrast, followed a similar analysis framework. To mitigate the risk of increased type 1 errors in post hoc comparisons, a two-stage step-up method of correction by Benjamini, Krieger, and Yekutieli was applied. A *p*-value adjusted to ≤0.05 was considered statistically significant. Spearman’s Rho was calculated to ascertain inter-rater agreement on subjective image quality, with r-values categorized as follows: 0–0.20 indicating negligible agreement, 0.21–0.40 weak, 0.41–0.60 moderate, 0.61–0.80 strong, and 0.81–1.00 very strong agreement.

## 3. Results

### 3.1. Study Population

**Figure 1:**
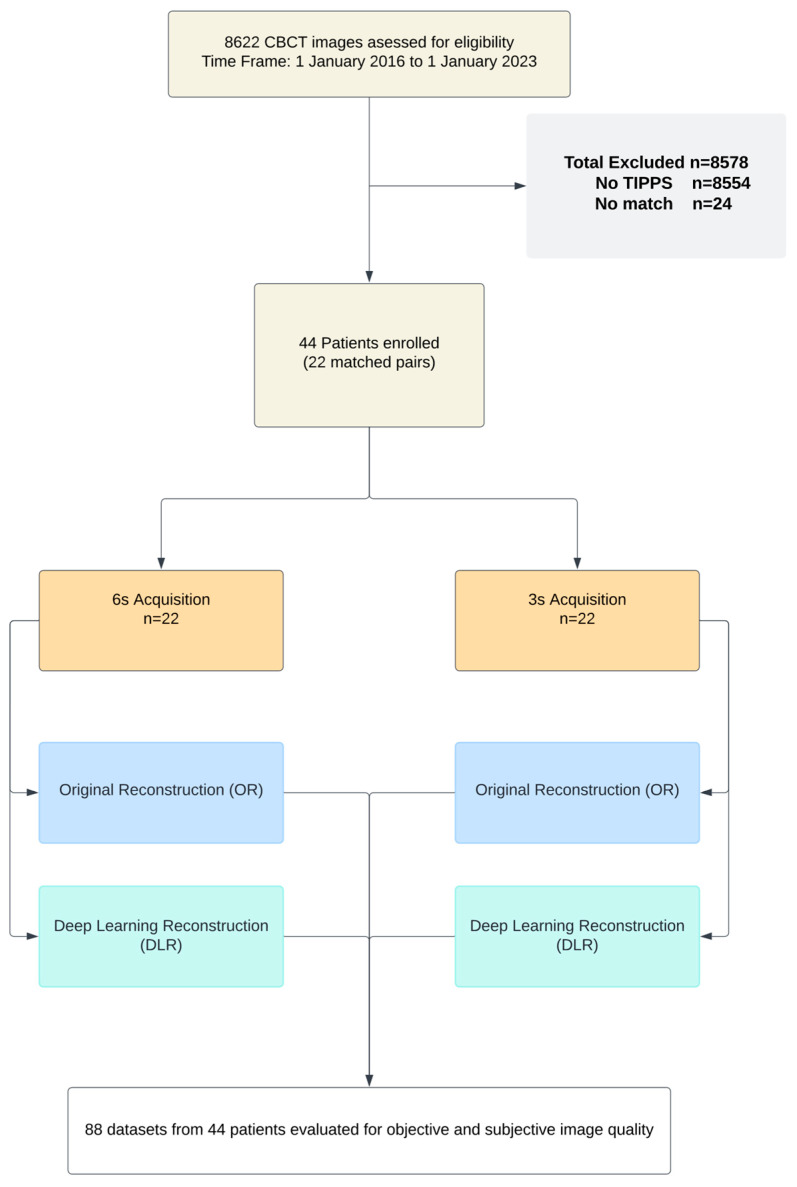
Study workflow.

Our experiment included 44 CBCT series from 22 matched patients who had undergone TIPSS at our institution. Overall, the patients had a mean height of 166.59 ± 16.16, a mean weight of 67.20 ± 17.92 kg, and a mean BMI of 23.80 ± 4.91 kg/m^2^. The mean overall DAP (Dose area product) of the 6 s series was 5138.50 ± 1325.57 µGy·m^2^, and the mean DAP of the 3 s series was 2514.06 ± 691.59 µGy·m^2^. For a graphical overview of the study’s workflow, see Figure 1, and for additional data on image acquisition and the study population’s metrics, refer to Table 1.

### 3.2. Objective Image Quality Assessment

**Figure 2:**
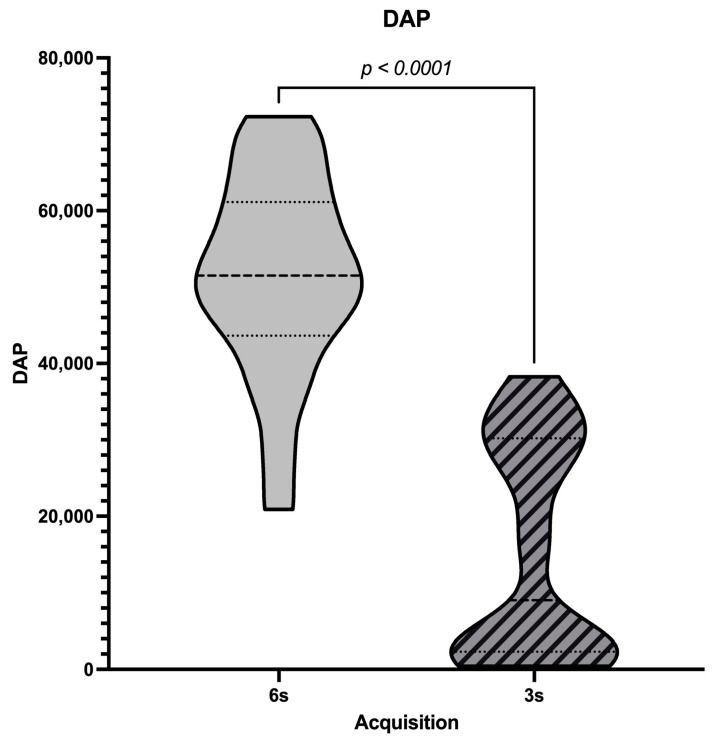
Correlation of CBCT Acquisition Duration with DAP.

In the objective image quality analysis, there were significant interactions between radiation dose and acquisition time (W = −253; *p* < 0.0001; Median difference = −38,073 µGy·m^2^; rs = 0.5562; *p* (one-tailed) = 0.0036; Figure 2).

**Figure 3:**
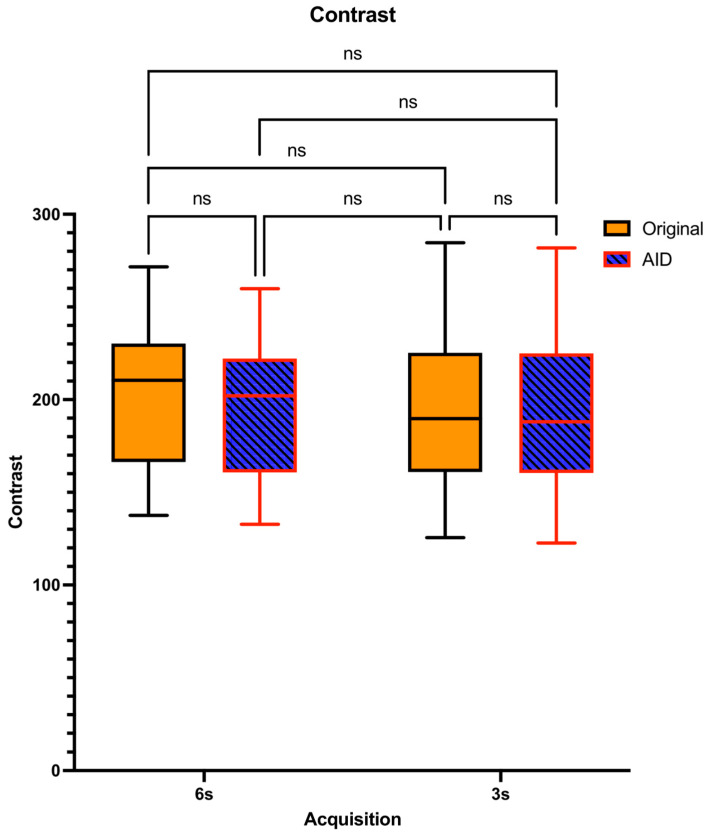
Distribution of Contrast Levels and Pairwise Comparisons Across Acquisition Times. ns = no statistically significant difference.

In our study’s objective image quality assessment, mixed-effects models showed significant interactions. The corrected post hoc comparisons of the Contrast showed no significant differences for the original and the denoised series for both the 6 s (*p* = 0.9554) and 3 s (*p* = 0.9998) acquisitions (Figure 3).

**Figure 4:**
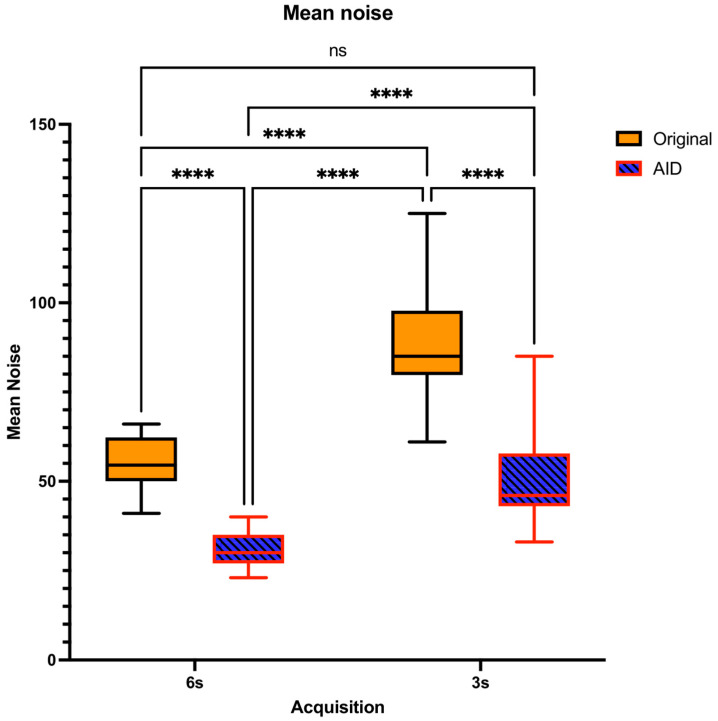
Distribution of Mean Noise Levels and Pairwise Comparisons Across Acquisition Times. ns = no statistically significant difference. **** = statistically significant difference.

The lowest noise levels were observed in the 6 s-AID series, succeeded sequentially by the 3 s-AID, 6 s-Original, and 3 s-Original series, with each transition demonstrating significant variations (*p* < 0.0001), except between the 6 s-Original and 3 s-AID series, where no significant difference was identified (*p* = 0.9423; Figure 4).

**Figure 5:**
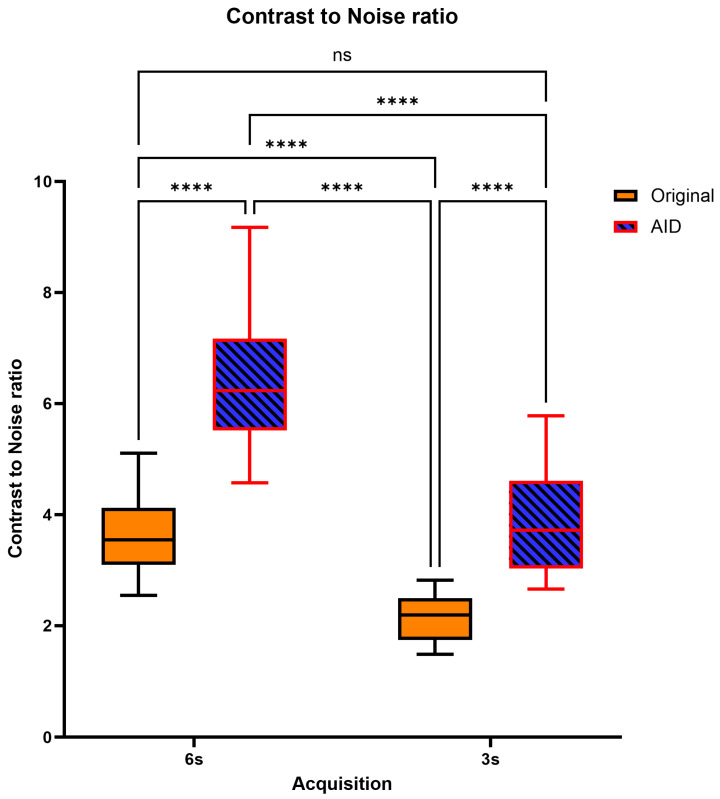
Distribution of CNR Levels and Pairwise Comparisons Across Acquisition Times. ns = no statistically significant difference. **** = statistically significant difference.

The highest CNR values were identified in the 6 s-AID series, followed in order by the 3 s-AID, 6 s-Original, and 3 s-Original series, all showing significant differences; however, there was no significant difference between the 6 s-Original and 3 s-AID series (*p* = 0.9968; Figure 5). Table 2 shows the mean scores ± standard deviation for the objective image analysis, along with the significance levels of the adjusted post hoc comparisons.

### 3.3. Subjective Image Quality Assessment

In the subjective analysis, there was a very strong agreement between raters, indicated by a Spearman’s Rho of 0.977 (each *p* < 0.001). Refer to Table 3 for the mean subjective scores ± standard deviation specific to each rater.

In the pooled comparisons for subjective image quality, Mixed Effects showed significant interactions. The 6 s AID series demonstrated the highest overall subjective image quality. No significant differences were observed between the 3 s AID and the 6 s Original series in terms of their overall subjective quality (*p* > 0.9999), both of which were superior to the 3 s Original dataset. The 3 s OR series was significantly rated as the lowest in overall subjective quality (*p* < 0.0001).

Consistent with the overall subjective image quality findings, the 6 s AID dataset achieved the highest ratings in contrast and sharpness (*p* < 0.0001). There was no significant difference between the 6 s Original and 3 s AID datasets in terms of contrast and sharpness (*p* > 0.9999). The 3 s Original dataset was rated significantly lower than all other groups for both contrast and sharpness (*p* < 0.0001).

In evaluating motion artifacts, both 3 s datasets were rated significantly higher, indicating fewer motion artifacts (*p* < 0.0001). No significant differences were observed between the Original and AID datasets at both 6 and 3 s (*p* = 0.981, *p* = 0.883).

**Figure 6:**
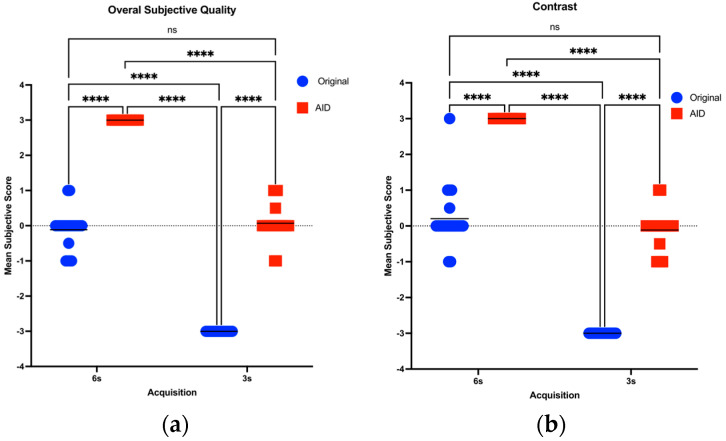

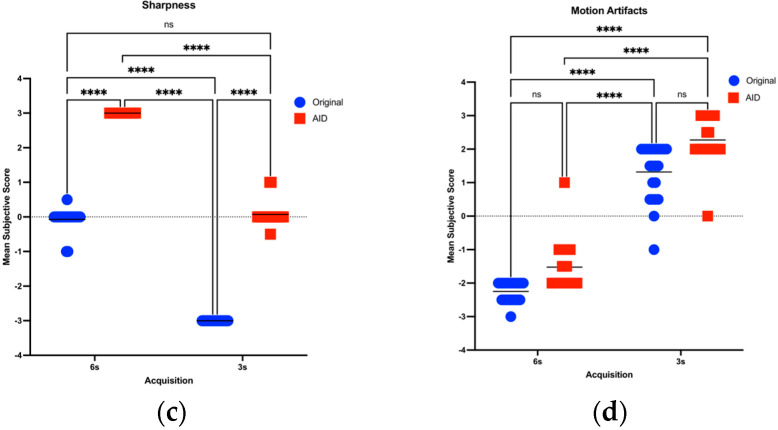
(**a**–**d**): Scatter dot plot of Mean Subjective Quality Scores Across 6 s and 3 s Acquisitions, assessing overall quality (**a**), contrast (**b**), sharpness (**c**), and motion artifacts (**d**). ns = no statistically significant difference. **** = statistically significant difference.

Figure 6 illustrates the data distribution and the ensuing post hoc comparative analyses and Table 4 presents the aggregated mean scores ± standard deviation for the subjective analyses, alongside the adjusted post hoc pairwise comparisons.

**Figure 7:**
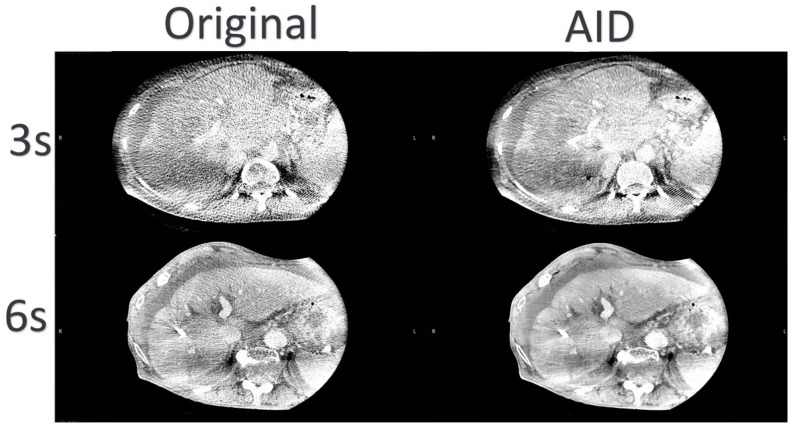
Representative TIPSS-CBCT Images: Noticeable Image Quality Enhancement with Minimized Noise and Motion Artifacts in the AID Series, Resulting in Superior Definition of the Portal vein.

Figure 7 displays comparative images illustrating enhanced delineation of the portal vein and liver contours, achieved through the reduction of noise and motion artifacts in the 3 s AID dataset in comparison to other datasets.

## 4. Discussion

The TIPSS procedure is emerging as a key intervention in the management of portal hypertension, a serious complication of CLD. However, accurate targeting of the portal vein during TIPSS can be challenging, compounded by the limitations of current imaging modalities. While cone-beam computed tomography (CBCT) provides the detailed cross-sectional images required for such procedures, it is hampered by a significant trade-off. Shorter acquisition times, while reducing patient exposure to radiation and minimizing respiratory motion artifacts, tend to increase image noise, potentially leading to non-diagnostic images. Conversely, longer acquisition times may improve image clarity but are prone to increased motion artifacts due to patient movement, potentially complicating the procedure.

The recent literature indicates that deep learning algorithms have the potential to improve CBCT image quality, enhancing diagnostic confidence by reducing noise and improving the contrast-to-noise ratio without the need for increased radiation doses or extended acquisition times [10,13,14]. Given these advancements, we hypothesized that an AI denoising algorithm could effectively optimize the trade-off between acquisition time and image quality in CBCT imaging for TIPSS.

In our objective image quality analysis, AID did not distort contrast across different acquisition times. Maintaining sufficient contrast is essential for precise and reliable diagnosis in CT imaging applications [15]. Furthermore, within the original reconstruction series, our study observed that shorter acquisition times correlated with diminished image quality, notably in the aspects of noise and the contrast-to-noise ratio. This observation is consistent with findings from a recent study on four-dimensional cone-beam computed tomography for image-guided lung cancer radiotherapy, which highlighted the adverse effects of very fast gantry rotations on image quality, particularly through increased streaking artifacts and reduced consistency in image metrics such as CNR and SNR when not employing motion-compensated reconstruction methods [16]. The AID series demonstrated superior objective image quality compared to the Original dataset across both acquisition times in our study. This superiority of AID in enhancing image quality, as evidenced by improved noise reduction and increased CNR, is in line with findings from recent studies. Choi et al. demonstrated that CNNs using self-supervised learning can enhance CBCT image quality by effectively denoising images without high-quality references, highlighting deep learning’s ability to improve noise reduction and CNR [14]. Additionally, a study by Yang et al. presented a transformer-based deep learning framework that effectively reduces the mean absolute error in CBCT images, highlighting its capability in precise CT number correction and noise/artifact reduction [17].

In accordance with the existing literature, our analysis noted a reduction in subjective image quality, including aspects of sharpness and contrast, associated with shorter acquisition times in the Original datasets [16,17,18]. Further comparison between the Original and AID datasets revealed a marked improvement in AID across these subjective quality indicators. These results align with a study by Zhao et al., which introduced a projection synthesis convolutional neural network to improve sparse-view CBCT images in image-guided radiotherapy, demonstrating significant quality improvements with lower imaging doses [19].

In the subjective motion artifact burden analysis, the data showed that the 3 s acquisition datasets, regardless of whether they were processed with Original or AID methods, exhibited fewer motion artifacts than the 6 s sets. This observation is corroborated by prior research, emphasizing the effectiveness of shorter acquisition times in diminishing motion artifacts [16,17].

Despite the development of deep learning techniques targeting motion artifacts in 4D CBCT—such as CNNs for missing projection prediction [20], residual U-shaped encoder-decoder network architecture for streak artifact reduction [21], and dual-encoder CNNs for improved edge sharpness [22]—our study employs a different AI-based CBCT reconstruction strategy. Our approach, focusing on shorter acquisition times, not only diminishes the presence of motion artifacts but also lowers patient radiation exposure. This strategy further decreases the risk associated with radiation while enhancing the overall quality of the imaging. Our study examined the impact of AI-denoising on CBCT images in TIPSS procedures, focusing on critical regions such as the right hepatic vein and the portal vein. However, since the radiologists evaluated these images without a ground truth reference, there is a risk that the AI-denoised images, despite appearing visually enhanced, might lack essential diagnostic details. The non-linear nature of AI-based denoising algorithms can introduce challenges in maintaining diagnostic accuracy, as these algorithms may inadvertently modify or obscure important features [23]. Cautious interpretation and further validation against ground truth are essential to ensure that these enhancements truly translate into improved outcomes for patients.

This study has limitations. First, as a single-center study, the results may have limited generalizability due to our institution’s specific patient demographics, treatment protocols, and technological resources. Second, the retrospective design of this study may have introduced biases in selection, data completeness, and classification, potentially affecting the robustness of our findings. Furthermore, due to the retrospective nature of this study, the AI-enhanced CBCT images were not utilized within the actual TIPSS procedure workflow. Although the AI denoising technique yielded significant improvements in image clarity, contrast, and noise reduction, these enhancements were not evaluated in a real-time clinical setting. Future research is needed to investigate the practical integration of AI-denoised CBCT images into the TIPSS procedure, assessing their impact on procedural efficiency, diagnostic accuracy, and patient outcomes. Third, the processing time for the AI denoising algorithm was not measured in this study. However, based on the U-net architecture employed, the processing time is likely minimal and would not interfere with clinical workflows. Nonetheless, future studies should consider measuring the processing time to confirm its negligible impact on the TIPSS procedure. Fourth, the lack of longitudinal data limits our ability to assess long-term outcomes or the effects of radiation exposure from the imaging techniques evaluated. In addition, the biometric matching of patients for comparison may not fully represent the diverse patient population. Subjective image quality assessments may introduce variability in interpretation. This study’s reliance on a specific deep learning-based reconstruction algorithm suggests that future technological developments could alter these findings. In addition, the use of proprietary software and hardware may limit the reproducibility of our results in different clinical settings.

In conclusion, AID significantly improved both objective and subjective CBCT image quality across varying acquisition times. By leveraging shorter acquisition times, our AI-based approach effectively reduced motion artifacts and minimized patient radiation exposure, underscoring its potential to enhance diagnostic precision and patient safety in clinical settings.

## Figures and Tables

**Table 1:** Image acquisition and the study population’s metrics.

Sex	Dataset	*n*	Age	Height	Weight	DAP	BMI
**Female**	3 s	9	40.89 ± 19.93	160.44 ± 8.13	62.44 ± 12.10	2368.11 ± 501.41	24.23 ± 3.88
	6 s	11	53.91 ± 16.78	165.82 ± 4.56	59.55 ± 7.61	4678.56 ± 644.74	21.67 ± 2.86
	Overall	20	48.05 ± 18.97	163.40 ± 6.80	60.85 ± 9.71	3638.86 ± 1309.73	22.82 ± 3.51
**Male**	3 s	13	54.23 ± 22.97	172.39 ± 18.72	70.80 ± 19.97	2615.11 ± 801.48	23.09 ± 4.36
	6 s	11	51.27 ± 20.45	165.55 ± 23.42	74.51 ± 23.81	5598.44 ± 1675.99	26.42 ± 6.87
	Overall	24	52.88 ± 21.43	169.25 ± 20.82	72.50 ± 21.41	3982.47 ± 1965.22	24.62 ± 5.77
**Overall**	3 s	22	48.77 ± 22.32	167.50 ± 16.17	67.38 ± 17.36	2514.06 ± 691.59	23.56 ± 4.12
	6 s	22	52.59 ± 18.31	165.68 ± 16.47	67.03 ± 18.87	5138.50 ± 1325.57	24.05 ± 5.68
	Overall	44	50.68 ± 20.26	166.59 ± 16.16	67.20 ± 17.92	3826.28 ± 1689.29	23.80 ± 4.91

DAP: Dose area product.

**Table 2:** Objective Metrics for Image Quality Assessment and Adjusted Two-Tailed Pairwise Comparisons.

Parameter	Dataset	Reconstruction	Mean ± SD	Adjusted Two-Tailed Pairwise Comparisons (*p*)			
				3 s-AID	3 s-Original	6 s-AID	6 s-Original
CNR	3 s	AID	3.81 ± 0.86	n/a	<0.0001	<0.0001	**0.9968**
		Original	2.15 ± 0.40	<0.0001	n/a	<0.0001	<0.0001
	6 s	AID	6.45 ± 1.23	<0.0001	<0.0001	n/a	<0.0001
		Original	3.65 ± 0.67	**0.9968**	<0.0001	<0.0001	n/a
Contrast	3 s	AID	190.99 ± 44.69	n/a	**>0.9999**	**>0.9999**	**0.9998**
		Original	191.91 ± 42.94	**>0.9999**	n/a	**>0.9999**	**>0.9999**
	6 s	AID	195.16 ± 35.88	**>0.9999**	**>0.9999**	n/a	**0.9554**
		Original	200.34 ± 38.67	**0.9998**	**>0.9999**	**0.9554**	n/a
Mean Noise	3 s	AID	51.23 ± 12.11	n/a	<0.0001	<0.0001	**0.9423**
		Original	89.64 ± 14.44	<0.0001	n/a	<0.0001	<0.0001
	6 s	AID	30.59 ± 4.60	<0.0001	<0.0001	n/a	<0.0001
		Original	55.18 ± 7.06	**0.9423**	<0.0001	<0.0001	n/a

Parameter: Contrast = Denoting the difference in Hounsfield Units (HU) between relevant areas; Mean noise = denoting mean noise; CNR = contrast-to-noise ratio; Datasets: 3 s = 3 seconds, 6 s = 6 seconds, AID = AI denoising; SD = standard deviation; *p* = denoting significance level. n/a = not applicable.

**Table 3:** Assessments Per Rater and Consistency Across Raters (Spearman’s Rho).

Parameter	Run	Reconstruction	Rater 1 Mean ± SD	Rater 2 Mean ± SD	Spearman’s Rho
Overall Subjective image quality	3 s	AID	0.011 ± 0.750	0.023 ± 0.758	0.990
		Original	−0.750 ± 0.435	−0.750 ± 0.435	1
	6 s	AID	0.750 ± 0.435	0.750 ± 0.435	1
		Original	−0.023 ± 0.758	−0.034 ± 0.765	0.990
Contrast	3 s	AID	−0.023 ± 0.758	−0.034 ± 0.765	0.990
		Original	−0.750 ± 0.435	−0.750 ± 0.435	1
	6 s	AID	0.750 ± 0.435	0.750 ± 0.435	1
		Original	0.045 ± 0.757	0.057 ± 0.764	0.990
Motion Artifacts	3 s	AID	0.557 ± 0.522	0.580 ± 0.519	0.954
		Original	0.330 ± 0.656	0.330 ± 0.690	0.888
	6 s	AID	−0.352 ± 0.644	−0.409 ± 0.600	0.839
		Original	−0.580 ± 0.496	−0.545 ± 0.501	0.794
Sharpness	3 s	AID	0.023 ± 0.727	0.011 ± 0.735	0.990
		Original	−0.750 ± 0.435	−0.750 ± 0.435	1
	6 s	AID	0.750 ± 0.435	0.750 ± 0.435	1
		Original	−0.023 ± 0.727	−0.011 ± 0.735	0.990

AID = AI denoising; SD = standard deviation;

**Table 4:** Subjective image quality metrics and adjusted two-tailed pairwise comparisons.

Parameter	Dataset	Recon	Mean ± SD	Adjusted Two-Tailed Pairwise Comparisons (*p*)			
				3 s AID	3 s Original	6 s AID	6 s Original
Overall Subjective image quality	3 s	AID	0.017 ± 0.752	n/a	<0.0001	<0.0001	**>0.9999**
		Original	−0.750 ± 0.435	<0.0001	n/a	<0.0001	<0.0001
	6 s	AID	0.750 ± 0.435	<0.0001	<0.0001	n/a	<0.0001
		Original	−0.028 ± 0.759	**>0.9999**	<0.0001	<0.0001	n/a
Contrast	3 s	AID	−0.028 ± 0.759	n/a	<0.0001	<0.0001	**0.9971**
		Original	−0.750 ± 0.435	<0.0001	n/a	<0.0001	<0.0001
	6 s	AID	0.750 ± 0.435	<0.0001	<0.0001	n/a	<0.0001
		Original	0.051 ± 0.758	**0.9971**	<0.0001	<0.0001	n/a
Motion Artifacts	3 s	AID	0.568 ± 0.515	n/a	**0.0751**	<0.0001	<0.0001
		Original	0.330 ± 0.656	**0.0751**	n/a	<0.0001	<0.0001
	6 s	AID	−0.381 ± 0.587	<0.0001	<0.0001	n/a	**0.1182**
		Original	−0.562 ± 0.472	<0.0001	<0.0001	**0.1182**	n/a
Sharpness	3 s	AID	0.017 ± 0.729	n/a	<0.0001	<0.0001	**0.9994**
		Original	−0.750 ± 0.435	<0.0001	n/a	<0.0001	<0.0001
	6 s	AID	0.750 ± 0.435	<0.0001	<0.0001	n/a	<0.0001
		Original	−0.017 ± 0.729	**0.9994**	<0.0001	<0.0001	n/a

Datasets: 3 s = 3 s, 6 s = 6 seconds, AID = AI denoising; SD = standard deviation, n/a = not applicable; *p* = denoting significance level.

## Data Availability

The data that support the findings of this study are available from University Hospital Tuebingen, but restrictions apply to the availability of these data, which were used under license for the current study and are not publicly available. However, the data can be made available from the authors upon reasonable request and with the permission of University Hospital Tuebingen and the approval of the Ethics Commission.

## Abbreviations

PH	Portal hypertension
CLD	Chronic liver disease
TIPSS	Transjugular intrahepatic portosystemic shunt
CBCT	Cone-beam CT
FOV	Field of view
CNR	Contrast-to-Noise Ratio
AI	Artificial Intelligence
BMI	Body mass index
AID	AI denoising
ROI	Regions of interest
HU	Hounsfield Units
SD	Standard Deviation
Original	Original reconstruction
DAP	Dose area product
CNN	Convolutional neural network
